# Heat Transfer Enhancement of Diamond Rib Mounted in Periodic Merging Chambers of Micro Channel Heat Sink

**DOI:** 10.3390/mi16050533

**Published:** 2025-04-29

**Authors:** Xin Lu, Lu Wang, Liangbi Wang, Yao Hu

**Affiliations:** 1 School of Mechanical and Electrical Engineering, Lanzhou Jiaotong University, Lanzhou 730070, China; luxing@mail.lzjtu.cn (X.L.); wanglu@mail.lzjtu.cn (L.W.); hy@mail.lzjtu.cn (Y.H.); 2 Key Laboratory of Railway Vehicle Thermal Engineering Ministry of Education, Lanzhou Jiaotong University, Lanzhou 730070, China

**Keywords:** micro channel heat sink, merging chambers, ribs, geometric parameter, heat transfer

## Abstract

The heat transfer enhancement of diamond-shaped ribs mounted in the periodic merging chambers of microchannel (MC) heat sinks is investigated using a numerical method for Reynolds number in the region of 300–700. Compared to triangular, rectangular, and cylindrical ribs, diamond-shaped ribs achieve 3.59%, 13.24%, and 6.34% higher enhancement effects, respectively, under the same mass flow rate. Further analysis of geometric parameters (length, width, and height) and rib positioning reveals that a rib height of *h*/*H*_ch_ = 0.8 provides optimal heat dissipation performance. For *Re* < 500, the optimal configuration is a rib length of *l*/*L*_merg_ = 0.55 and a width of *b*/*W*_ch_ = 0.8, while for 500 < *Re* < 700, it shifts to *l*/*L*_merg_ = 0.36 and *b*/*W*_ch_ = 1.6. For *s*/*L*_merg_, the smaller it is, the shorter the main flow separation time, thereby improving heat transfer efficiency.

## 1. Introduction

As science and technology continue to advance, electronic components are becoming increasingly lightweight, intelligent, and miniaturized. The growing cooling demands of micro electromechanical systems (MEMSs) require heat sinks to also be miniaturized in order to effectively dissipate the heat generated by microelectronic components. Without efficient and modern cooling systems, the rapid development of MEMSs will be hindered, potentially leading to overheating and system failures. Microchannels (MCs), compared to traditional heat sinks, feature a smaller size and larger heat transfer surface area. They are widely used in MEMSs, effectively enhancing cooling efficiency and ensuring the safe operation of microelectronic devices. Therefore, optimizing and improving the performance of MC cooling systems has become a key focus for researchers, as it is crucial for improving the reliability and safety of MEMS devices.

Li et al. [1] presented an innovative microchannel heat sink that combines rectangular and cylindrical ribs. They used three-dimensional numerical simulations to assess how various arrangement patterns, along with the diameter and height of the ribs, affect hydrothermal performance and temperature uniformity. Yin et al. [2] investigated the thermal efficiency of a rectangular microchannel heat exchanger that includes rectangular fins. Their numerical simulations analyzed how offset distances, fin orientations, and elevations affect performance. The results showed that altering the configuration and arrangement of the fins enhances thermal transfer efficiency and fluid mixing, improving heat dissipation. Du et al. [3] conducted a study on the thermal and fluidic characteristics of diamond microchannel heat sinks featuring different types and shapes of orthogonal ribs. They significantly improved thermal transfer efficiency by disrupting fluid flow and increasing the solid–liquid contact area. The orthogonal rib structure showed better heat conduction and flow uniformity, outperforming traditional designs. This research offers a solid solution and conceptual foundation for managing the thermal performance of high-power electronic devices. Alfellag et al. [4] presented a plate-fin MC heat sink with trapezoidal cavities and elliptical fins, using numerical analysis to evaluate the effects of the elliptical fin aspect ratio, cavity center position, and inclined groove width on cooling performance. They found that when the aspect ratio was 1.25, the values of the Nusselt number, drag coefficient, and performance assessment metric were optimal.

Ma et al. [5] designed two types of silicon MCs featuring periodic injection and throttling structures, investigating their heat transfer characteristics through experiments. Their results revealed that while the throttle-shaped MC experienced a significant increase in resistance compared to the jet-shaped MC, it also demonstrated markedly enhanced heat transfer capacity. This enhancement is attributed to the periodic throttling, which blocks fluid flow and induces back flow, promoting continuous remixing of the fluid and improving heat dissipation. Vasilev et al. [6] investigated how the diameter, configuration, and placement of circular pin fins affect fluid dynamics and thermal characteristics in microchannel (MC) heat sinks under laminar flow conditions. They analyzed parameters such as pressure drop, Nu, and thermal resistance for circular pin fins with varying diameters, spacings, and heights within the microchannel. The findings were then compared to those of conventional microchannels without rib structures. Ultimately, they determined the most effective shape and arrangement for the circular pin fins. Tian et al. [7] proposed a novel MC structure with a variable-width merging chamber to address uneven flow distribution within the MC. This design promoted uniform fluid mixing in adjacent channels, resulting in a 37.5% improvement in flow uniformity. Li et al. [8] introduced an MC structure with concave holes on the sidewalls and pin fins at the center, aiming to improve thermal transfer through periodic flow disturbances. Heat resistance and enhanced heat transfer factors were utilized to assess the cooling efficacy. The findings indicated that the relative length of the cavity’s bottom edge had a notable effect on thermal dissipation, emphasizing the importance of periodic spoiler structures in improving cooling efficiency. Pourhammati and Hossainpour [9] and Lin et al. [10,11] conducted numerical studies on fluid flow patterns and thermal performance in wavy MC heat sinks, and further improved heat dissipation by adjusting the relative amplitude and wavelength along the MC.

Many researchers have introduced modifications to the continuous structure of MCs by incorporating periodic merging chambers, dividing the channels into distinct flow regions, and introducing different rib configurations within the merging chambers to facilitate flow expansion, separation, disruption, and re-contraction [12,13,14]. Ansari et al. [12] introduced a hybrid microchannel-pin fin heat sink aimed at improving thermal performance. This design includes rectangular microchannels in areas with low heat flux and cylindrical ribs in high heat flux regions, creating a periodically merged chamber structure known as PMMC-C. The study evaluated key performance metrics such as thermal resistance, pumping power, temperature consistency, and maximum temperature increase at the hotspot. In comparison to conventional non-hybrid heat sinks, the hybrid design demonstrated significant improvements in thermal performance. Chai et al. [13] explored the periodically merged chamber rectangular MC mounted rectangular rib structure (PMMC-R), varying their dimensions and placements to assess their influence on pressure drop and heat transfer, ultimately identifying optimal configurations. Wong et al. [14] focused on PMMC mounted triangular rib structure (PMMC-T), examining how variations in length, width, and height influenced radiator performance to identify the best structural parameters.

**Figure 1:**
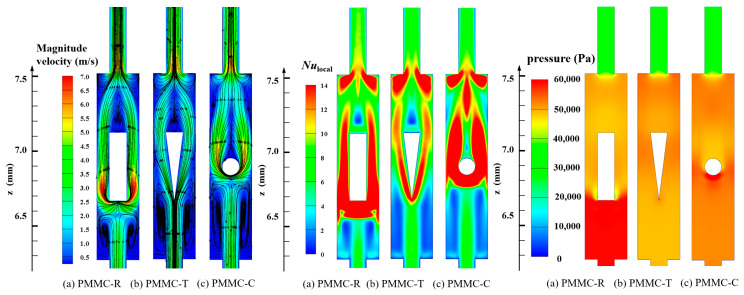
Flow field, *Nu*_local_, and pressure field of different MCs in the *y* = 0.15 mm plane at *Re* = 500.

The above review indicates that existing ribs arranged in merging chambers include rectangular, triangular, and cylindrical shapes. As shown in Figure 1, these ribs generally cause a large recirculation zone at their rear, where the heat transfer coefficient is lower, back pressure is reduced, and form drag is higher. Figure 1 also provides some quantitative evaluation of these shortages.

Motivated by these facts, this study tries to explore some new rib designs, to decrease the recirculation zone, to increase the heat transfer in the rear of the fin, to increase the pressure on the rib back, and then to further optimize the heat transfer performance of MC. To realize this goal, the heat transfer performances of the MC with the periodically merged chambers having mounted diamond ribs are investigated. The detailed heat transfer performances of the MC mounted with the diamond ribs are presented. Compared to the performances of the triangular ribs, the rectangular ribs, and the cylindrical ribs, the diamond-shaped ribs achieve heat transfer enhancement performances of 3.59%, 13.24%, and 6.34%, respectively, under the same mass flow rate constraint.

## 2. Physical Model

### 2.1. Geometric Model

**Figure 2:**
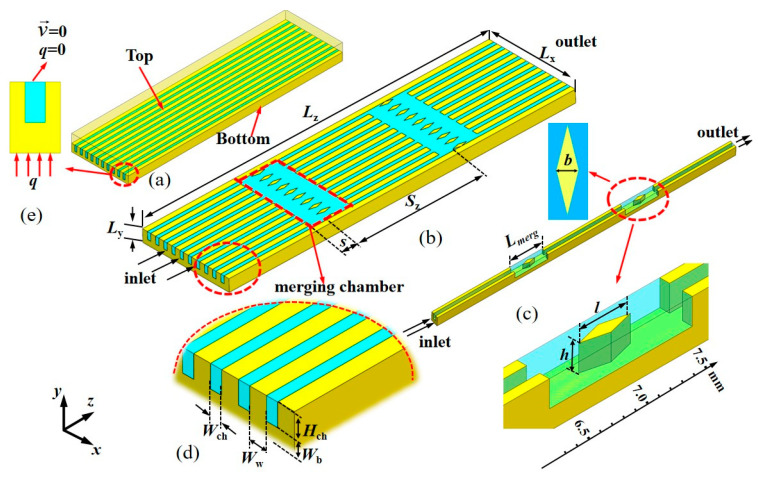
MC studied: (**a**) the overall structure of MC; (**b**) the structure with merged chambers in the continuous channel; (**c**) the single-channel computational model; and (**d**) the local parameter structure of MC; (**e**) the main view direction of the inlet.

The MC heat sink studied is shown in Figure 2, with overall dimensions of *L*_z_ = 10 mm (length), *L*_x_ = 2.5 mm (width), and *L*_y_ = 0.35 mm (height).

Figure 2a depicts a continuous MC (CMC), also known as the traditional MC. Figure 2b illustrates the design of two periodically merged chambers within the continuous rectangular MC, where fins are installed. Water, as the cooling medium, flows through the channel in the *z* direction. To reduce computational load, this study uses a single channel as the computational model. The channel has a width (*W*_ch_) of 0.1 mm and a height (*H*_ch_) of 0.2 mm, as illustrated in Figure 2c,d. Dimensions of the MC are in Table 1.

### 2.2. Mathematical Model

The material selected for the MC heat sink under study is silicon; the physical parameters of silicon are shown in Table 2. As shown in Figure 2e, a uniform heat flux is imposed on the bottom surface of the heat sink, while on the top surface, no slip velocity and thermal insulation boundary conditions are applied. Water circulates through the internal channels as the cooling medium.

To check whether the continuum mechanics theory can be used, the Knudsen number (*Kn*) is determined. *Kn* represents the ratio between the average free path of molecules and the system’s representative length, measuring the typical distance a molecule travels prior to collisions in relation to the representative length of the system [15]. *Kn* is calculated as follows:

(1) Kn=λ/L

In the above equation, *λ* refers to the molecular mean free path, which for water in liquid state is typically about 3 × 10^−10^ m [16,17]. *L* refers to the smallest size of the control volume, and for the MC structure discussed in the text, the characteristic length is *W*_ch_/(the number of the grid points along the x direction), that is 0.1 mm/20 = 5 × 10^−6^ m. Therefore, the maximum value of *Kn* is 3 × 10^−10^/5 × 10^−6^ = 6 × 10^−5^, which is much smaller than the critical value of 0.01 [18]. Thus, the continuum mechanics theory is generally considered applicable.

Under the specified conditions, the flow within the MC is assumed to be steady, incompressible, and laminar. A constant heat flux is applied at the bottom of the MC. Based on these assumptions, the governing equations for the conservation of mass and momentum are as follows:

Conservation of mass:

(2) $$\frac{\partial u}{\partial x}+\frac{\partial v}{\partial y}+\frac{\partial w}{\partial z}=0$$

where *u*, *v*, and *w* represent the velocity components of the fluid in the *x*, *y*, and *z* directions, respectively.

The momentum conservation equations in the *x*, *y*, and *z* directions are given as follows, respectively:

*x*-direction:

(3) $$\frac{\partial u}{\partial t}+u\frac{\partial u}{\partial x}+v\frac{\partial u}{\partial y}+w\frac{\partial u}{\partial z}=-\frac{1}{\rho }\frac{\partial p}{\partial x}+\nu \left(\frac{\partial^{2}u}{\partial x^{2}}+\frac{\partial^{2}u}{\partial y^{2}}+\frac{\partial^{2}u}{\partial z^{2}}\right)$$

*y*-direction:

(4) $$\frac{\partial v}{\partial t}+u\frac{\partial v}{\partial x}+v\frac{\partial v}{\partial y}+w\frac{\partial v}{\partial z}=-\frac{1}{\rho }\frac{\partial p}{\partial y}+\nu \left(\frac{\partial^{2}v}{\partial x^{2}}+\frac{\partial^{2}v}{\partial y^{2}}+\frac{\partial^{2}v}{\partial z^{2}}\right)$$

*z*-direction:

(5) $$\frac{\partial w}{\partial t}+u\frac{\partial w}{\partial x}+v\frac{\partial w}{\partial y}+w\frac{\partial w}{\partial z}=-\frac{1}{\rho }\frac{\partial p}{\partial z}+\nu \left(\frac{\partial^{2}w}{\partial x^{2}}+\frac{\partial^{2}w}{\partial y^{2}}+\frac{\partial^{2}w}{\partial z^{2}}\right)$$

where *t* represents time, *ρ* is the fluid density (constant), *p* is pressure, and *ν* is kinematic viscosity.

Energy equation:

(6) $$\rho c_{p\;}\left(u\frac{\partial T}{\partial x}+v\frac{\partial T}{\partial y}+w\frac{\partial T}{\partial z}\right)=k_{f}\left(\frac{\partial^{2}T}{\partial x^{2}}+\frac{\partial^{2}T}{\partial y^{2}}+\frac{\partial^{2}T}{\partial z^{2}}\right)\;\;\;\;(\mathrm{for}\mathrm{the}\mathrm{fluid})$$

(7) $$\frac{\partial^{2}T}{\partial x^{2}}+\frac{\partial^{2}T}{\partial y^{2}}+\frac{\partial^{2}T}{\partial z^{2}}=0\;\;(\mathrm{for}\mathrm{substrate}\mathrm{conduction})$$

where *T* represents temperature, *c_p_* is the fluid’s specific heat capacity at constant pressure, and *k_f_* is the fluid’s thermal conductivity.

### 2.3. Boundary Condition

At *z* = 0, for the MC inlet related to the fluid,

(8) $$w=w_{in},\;u=v=0,\;T_{f}=T_{in}=293K$$

for solid surface,

(9) $$\partial T_{s}/\partial z=0$$

At *z* = 10 mm, for the MC outlet related to the fluid,

(10) $$u=v=0,\;p_{f}=p_{\mathrm{out}}=0,\;\frac{\partial w}{\partial z}=0,\;\frac{\partial T_{f}}{\partial z}=0$$

for solid surface,

(11) $$\partial T_{s}/\partial z=0$$

At walls (*x* = 0 and *x* = 0.25 mm):

(12) $$\frac{\partial T_{s}}{\partial x}=0$$

To make validation of the present results easy, at the bottom of MC (*y* = 0), a uniform heat flux is specified as in [13,14]:

(13) $$q=1.22\;\mathrm{MW}\cdot m^{-2}$$

At the top of MC (*y* = 0.35 mm):

(14) $$u=v=w=0,\frac{\partial T_{f}}{\partial y}=\frac{\partial T_{s}}{\partial y}=0$$

At the fluid–solid interface:

(15) $$u=v=w=0,T_{s}=T_{f},-k_{s}\frac{\partial T_{s}}{\partial n}=-k_{f}\frac{\partial T_{f}}{\partial n}$$

The average friction coefficient is

(16) $$f=\frac{2\Delta pD_{h}}{L\rho w_{m}^{2}}$$

The Reynolds number is expressed as

(17) $$Re=\frac{\rho w_{m}D_{h}}{\mu }$$

The formula for the characteristic length is

(18) $$D_{h}=\frac{4A}{P}=\frac{2(W_{\mathrm{ch}}\times H_{\mathrm{ch}})}{\left(W_{\mathrm{ch}}+H_{\mathrm{ch}}\right)}$$

The local heat transfer coefficient is defined as

(19) $$h_{\mathrm{local}}=\frac{q_{w}(x,y,z)}{\left(T_{w}(x,y,z)-\bar{T_{f}}\right)}$$

The average heat transfer coefficient is given by

(20) $$\bar{h}=\frac{qA_{b}}{A_{\mathrm{contact}}(\bar{T_{w}}-\bar{T_{f}})}$$

The fluid temperature’s mass-weighted average is expressed as

(21) $$\bar{T_{f}}=\frac{\int \rho (x,y,z)T_{f}(x,y,z)\cdot dV}{\int \rho (x,y,z)\cdot dV}$$

The average Nusselt number is defined as follows:

(22) $$Nu=\frac{\bar{h}D_{h}}{k}$$

The local Nusselt number is defined as follows:

(23) $$Nu_{\mathrm{local}}=\frac{h_{local}D_{h}}{k}$$

The span-averaged Nusselt number:

(24) $$Nu_{s}\left(z\right)=\int_{S_{p}}Nu_{\mathrm{local}}\Delta zds/\int_{S_{p}}\Delta zds$$

The absolute value of the vortex flux in the mainstream direction is

(25) $$J_{\mathrm{ABS}}^{n}=\left|\omega^{n}\right|$$

When the main direction coincides with the *z* direction, *ω*^n^ can be expressed by the following equation:

(26) $$\omega^{n}=\frac{\partial v}{\partial x}-\frac{\partial u}{\partial y}$$

The secondary flow characteristic velocity *U*_s_ is

(27) $$U_{s}=D_{h}J_{\mathrm{ABS}}^{n}$$

The dimensionless number *Se* represents the strength of secondary flow and is expressed as

(28) $$Se=\rho D_{h}U_{s}/\mu$$

The span-averaged secondary flow intensity *Se*_s_ over the cross-sectional area is

(29) $$Se_{s}\left(z\right)=\frac{\rho D_{h}}{\mu }\int_{A}U_{s}dA/\int_{A}dA$$

The volumetric averaged secondary flow intensity *Se*_v_ over the flow field is

(30) $$Se_{v}=\frac{\rho D_{h}}{\mu }\int_{V}U_{s}dV/\int_{V}dV$$

With the same mass flow rate constraint, the heat transfer enhancement factor (*JF*) is described as the ratio between the average *Nu* of the merged chamber microchannel (MC) featuring various rib structures and the average *Nu*_0_ of the PMMC. It is formulated as follows:

(31) $$JF=\frac{Nu/Nu_{0}}{f/f_{0}}$$

## 3. Validation of Numerical Method

### 3.1. Mesh Independence and Numerical Model Validation

The governing equations were solved using the finite volume-based computational fluid dynamics solver FLUENT 17.0. The SIMPLE method was employed for coupling the pressure and velocity fields. A second-order upwind scheme was used for the convective terms, while a second-order central difference scheme was applied for the diffusive terms. The algebraic equations were solved using the algebraic multigrid (AMG) method. The solutions were considered to be converged when the normalized residual values of all variables were less than 10^−7^, except for the energy equation, where it was 1 × 10^−8^. To guarantee result accuracy, a grid independence study and validation of the numerical simulation method were performed. Choosing an appropriate grid configuration is essential for accurately simulating the thermal performance of the MC heat sink. The number of grid elements significantly affects the simulation results. To confirm grid independence, various grid densities were tested using the conventional rectangular MC model. This analysis was carried out for a structure with a heating power of 1.22 MW/m^2^, an inlet velocity of *w*_in_ = 3.84 m/s, and an inlet temperature of *T*_in_ = 293 K. The findings are summarized in Table 3.

We conducted a grid independence study to ensure that the computational results were not affected by the grid size. Various grid resolutions were tested, and the results for key output parameters (in terms of ${\bar{T}}_{f}$, *Nu*, and *f*) were compared to confirm that further grid refinement only caused minimal changes in the results. When the differences in results were smaller than a specified threshold (1%), the grid was considered sufficiently refined. During the numerical simulations, we ensured that the residuals of the continuity and energy equations decreased to at least 10^−8^ as the convergence criterion. In Table 3, the results of schemes 2 and 3 (in terms of ${\bar{T}}_{f}$, *Nu*, and *f*) are compared with those of scheme 1. The differences between the results of scheme 2 and scheme 1 are significantly smaller than the threshold of 1%, and scheme 2 features a moderate grid count. Therefore, after balancing accuracy and computational efficiency, scheme 2 is chosen for the numerical simulation of the microchannel heat sink.

### 3.2. Theoretical Verification

**Figure 3:**
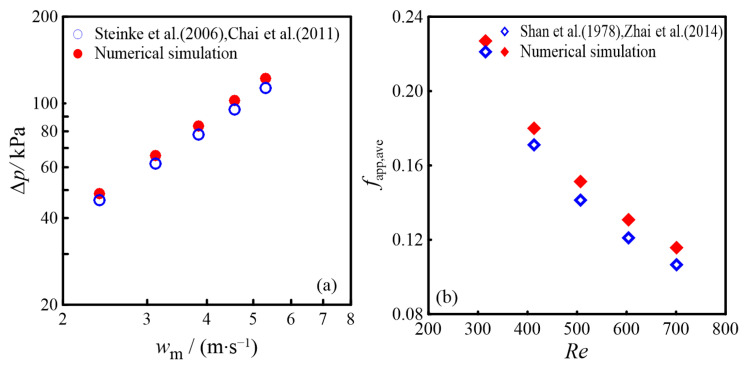
Comparative analysis of numerical and theoretical results: (**a**) Δ*p* obtained by Steinke et al. [19] and Chai et al. [20]; (**b**) *f_app,ave_* obtained by Shan et al. [21] and Zhai et al. [22].

The studies in [19,20] proposed a formula to compute the pressure drop in CMCs:

(32) $$\Delta p=\frac{fRe\mu w_{m}L}{2D_{h}^{2}}+K\frac{\rho w_{m}^{2}}{2}$$

(33) $$fRe=96\left(1-1.3553\alpha_{c}+1.9467\alpha_{c}^{2}-1.7012\alpha_{c}^{3}+0.9564\alpha_{c}^{4}-0.2537\alpha_{c}^{5}\right)$$

(34) $$K=0.6797+1.2197\alpha_{c}+3.3089\alpha_{c}^{2}-9.5921\alpha_{c}^{3}+8.9089\alpha_{c}^{4}-2.9959\alpha_{c}^{5}$$

where *α*_c_ represents the aspect ratio of width to height, *w*_m_ is the fluid’s average velocity, m/s. When *w*_m_ ranges from 2.2 m/s to 5.3 m/s, Figure 3a presents both the pressure drop obtained from numerical simulation results for the CMC and those derived from Equation (32). As shown in Figure 3a, the numerical simulation results closely align with the analytical calculations, with a maximum deviation of 5.76%.

References [21,22] proposed the following formula for determining the friction coefficient in the CMC under laminar flow conditions:

(35) $$f_{\mathrm{app},\mathrm{ave}}Re=\sqrt{{\left[\frac{3.2}{L/(D_{h}Re)}\right]}^{2}+{\left(fRe\right)}^{2}}$$

where *f*_app,ave_ is the Darcy friction coefficient.

Figure 3b compares the numerical findings of the Darcy friction coefficient in the CMC against the analytical calculations. The comparison reveals that the numerical results align closely with the analytical calculations, showing a maximum discrepancy of 6.45%. This demonstrates the reliability of the numerical method used to evaluate the pressure drop and friction coefficient in the CMC. The error analysis indicates that the potential sources of error may come from the following aspects: firstly, the analytical calculations are based on certain assumptions and simplifications, neglecting some actual variation characteristics; secondly, the limitations of numerical simulation accuracy, such as grid discretization, convergence criteria, and time step settings. Overall, the error range in Figure 3a,b is within an acceptable range.

### 3.3. Experimental Verification

**Figure 4:**
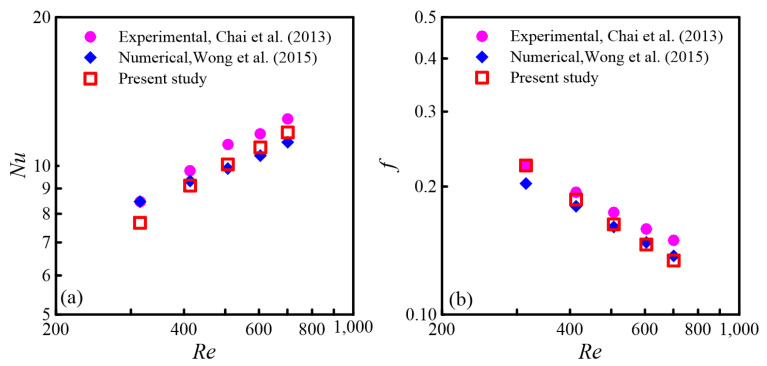
Analysis of numerical calculations [14] and experimental findings [13]: (**a**) *Nu*; (**b**) *f*.

To further validate the precision of the numerical results, the same structure as the experimental model by Chai [13] was used for numerical calculations. It is worth noting that Wong [14] also validated the same structure through numerical simulations. *Nu* and *f* obtained from the numerical calculations were contrasted with both the experimental data and the numerical findings of Wong [14]. The comparison is shown in Figure 4a,b.

From Figure 4a,b, it can be concluded that the average *Nu* and *f* obtained from the numerical calculations in this paper are very close to the experimental results and Wong’s numerical results [14]. Comparing with the experimental data, Figure 4a shows that the maximum deviation for the average *Nu* is 9.48%, while Figure 4b indicates that the maximum deviation for the average *f* is 10.47%. Similarly, the errors mainly originate from the measurement accuracy of the experiments and the fluctuations in experimental conditions. For the *Nu* and *f* values, the error is approximately around 10%, which is within an acceptable range. These results further confirm the reliability of the numerical method used for the CMC. Subsequently, the same numerical calculation method was applied to MC models with different rib shapes in the merging chambers, ensuring the reliability of the simulation outcomes.

## 4. Results and Discussion

### 4.1. The Local Characteristics

#### 4.1.1. Velocity Fields

**Figure 5:**
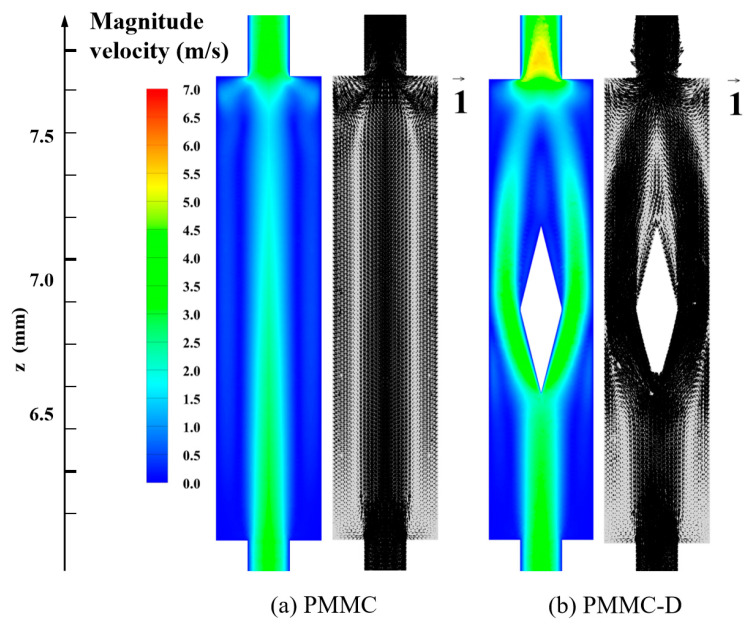
Flow field distributions of different MCs in *y =* 0.20 mm plane at *Re* = 500.

To analyze the thermal characteristics of the novel microchannel (MC) model with merged chambers and rib combinations, it is essential to first investigate the fluid flow characteristics within the channel. Figure 5 illustrates the streamline patterns and velocity distributions for the PMMC and PMMC-D designs at *y =* 0.20 mm and *Re* = 500. In the PMMC design, when the main flow enters the merged microchamber, the fluid velocity decreases on both sides, while the central flow impacts the bottom of the microchamber, forming two uneven vortices within the chamber. After introducing diamond-shaped ribs into the PMMC design, the main flow impacts the rib structure upon entering the merged chamber, generating backflow near the wall surfaces. This backflow exhibits a symmetrical distribution. Due to the conical shape of the diamond ribs at the front and rear, the fluid converges smoothly toward the outlet, resulting in a smaller recirculation region. Downstream, the backflow velocity decreases and re-merges with the main flow.

**Figure 6:**
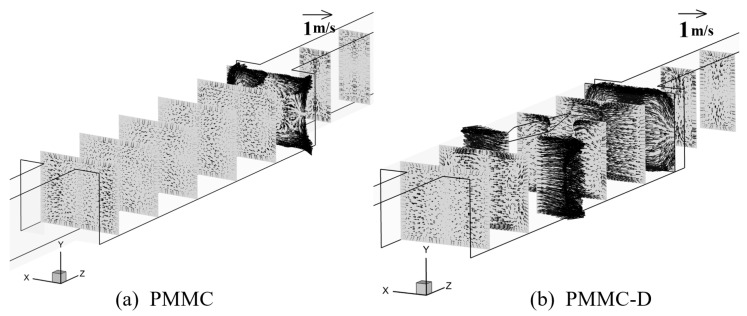
Velocity fields on different cross-sections at *Re* = 500.

To better analyze the fluid flow in PMMC and PMMC-D, Figure 6 presents the velocity vectors of the fluid on the cross-section within the microchamber. Comparing PMMC and PMMC-D, in PMMC, vortices are only generated at the bottom of the microchamber as the fluid impacts the front wall of the chamber. In contrast, the presence of diamond-shaped ribs alters the flow direction, velocity, and vortex rotation. According to the Bernoulli effect, at the widest cross-section of the rib, the fluid velocity and longitudinal vortices are most pronounced. The diamond-shaped ribs promote efficient fluid mixing, leading to continuous boundary layer disruption and redevelopment, thereby enhancing heat transfer performance.

#### 4.1.2. Temperature Fields

**Figure 7:**
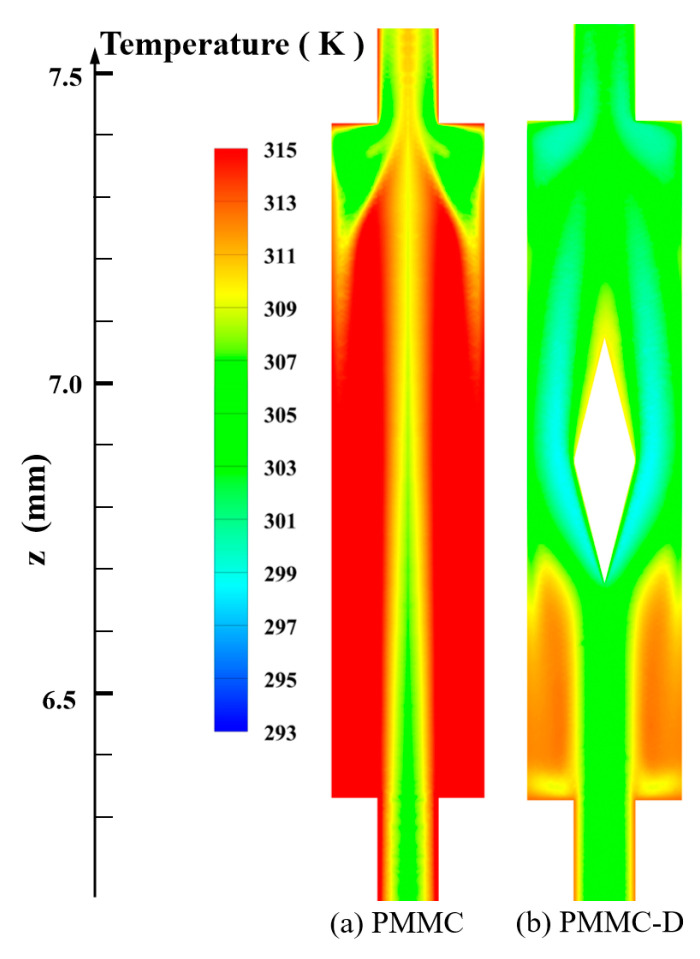
Temperature fields of different MCs in *y =* 0.15 mm plane, at *Re* = 500.

Figure 7 shows the temperature field distribution of two structures under the same conditions. Compared to PMMC, PMMC-D exhibits significantly different temperature distributions, with lower solid temperatures, highlighting the critical influence of diamond-shaped ribs on the temperature field.

**Figure 8:**
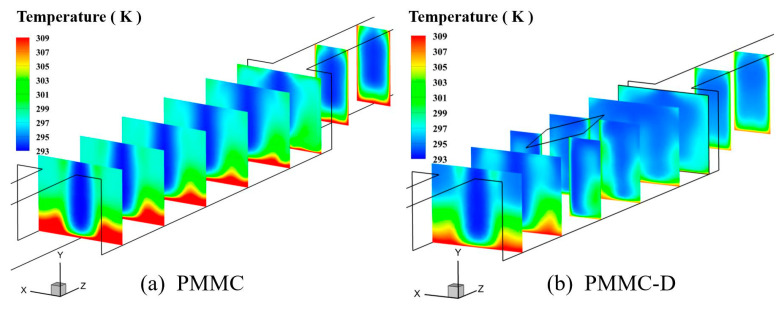
Temperature fields on different cross-sections at *Re* = 500.

Diamond-shaped ribs promote enhanced mixing between the mainstream and the backflow, leading to a significant temperature gradient on the heated walls of the MC. This gradient peaks around the ribs and in the gaps between the ribs, while the downstream region shows lower temperatures, indicating improved heat conduction on both the merged chamber and rib surfaces. In contrast, PMMC exhibits smaller changes in the temperature field, with a thicker thermal boundary layer and higher temperatures at the fluid–solid interface. To provide a clearer observation, different cross-sections within the microchamber were taken, and their temperature distributions are shown in Figure 8.

Figure 8a,b show the temperature distributions at different cross-sections within the PMMC and PMMC-D microchannels, respectively. Comparing the temperatures at different cross-sections, it is observed that in PMMC, the temperature at the fluid–solid interface is higher, and the temperature variation across the cross-section is relatively small. In contrast, in PMMC-D, after the fluid passes through the diamond-shaped ribs, the enhanced fluid mixing promotes momentum and heat exchange between the mainstream and near-wall fluids, leading to a significant decrease in temperature at the fluid–solid interface, thereby greatly enhancing the thermal performance of this heat sink.

#### 4.1.3. Local Nusselt Number

**Figure 9:**
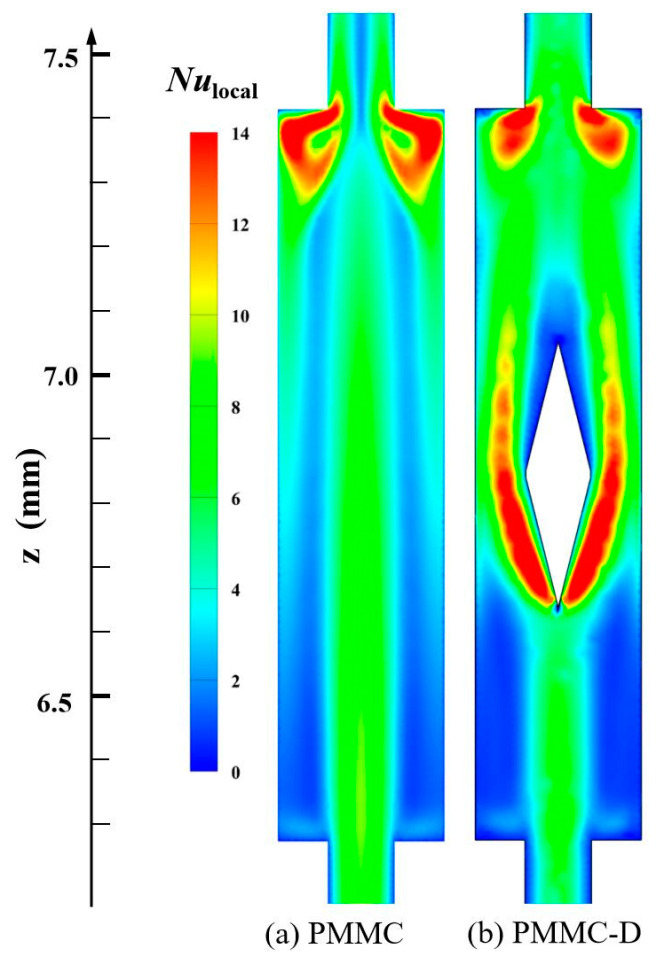
*Nu*_local_ of different MCs in *y =* 0.15 mm plane, at *Re* = 500.

Figure 9 illustrates the *Nu*_local_ value distributions of PMMC and PMMC-D on the *y* = 0.15 mm plane at *Re* = 500. The local Nusselt number (*Nu*_local_) is based on *h*_local_ defined by Equation (19). Figure 9 shows the differences in heat transfer performance on the surface at different positions. In Figure 9a, the highest *Nu*_local_ values are mainly located in areas where the fluid transitions from the microchamber into the MC, causing a sudden decrease in cross-sectional area and fluid impingement on the ribs. In Figure 9b, the mainstream is interrupted by the diamond-shaped ribs, and the fluid is directed to both sides of the ribs, leading to a thinning of the thermal boundary layers on both sides and higher *Nu*_local_ values in these regions. Behind the diamond-shaped ribs, the *Nu*_local_ value decreases due to the formation of recirculation zones. Additionally, before the fluid continues to flow from the merged microchamber into the MC, the cross-sectional area suddenly decreases, causing the fluid to impinge on the ribs between adjacent channels. The fluid is diverted to both sides, and its velocity increases, resulting in a higher intensity of *Nu*_local_ in these regions.

#### 4.1.4. Span Local Nusselt Number

**Figure 10:**
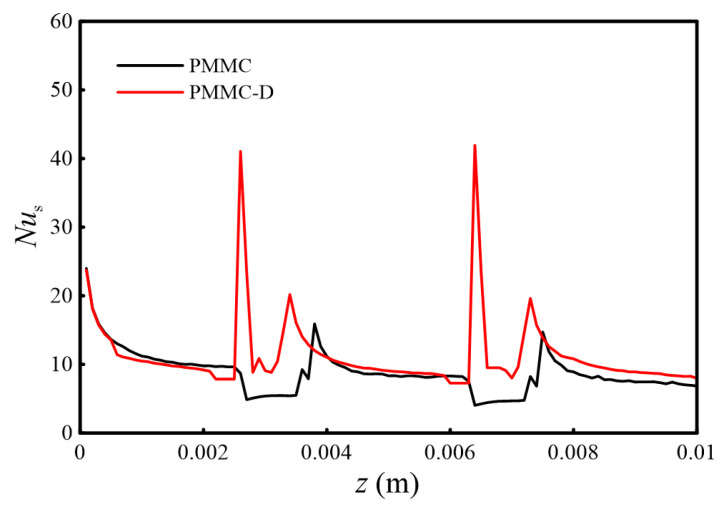
Span-averaged *Nu*_s_ along the main flow direction.

Figure 10 shows the span-averaged Nusselt numbers (*Nu*_s_) for PMMC and PMMC-D in the direction of the main flow.

From Figure 10, it is observed that due to the inlet effect, there is a higher *Nu*_s_. As the flow progresses, the boundary layer continues to thicken, leading to a gradual weakening of the inlet effect and a decrease in *Nu*_s_. Observing the *Nu*_s_ value of PMMC-D, when the fluid enters the merged microchamber and reaches the vortex generator, the shear force increases, resulting in a higher *Nu*_s_ value. In contrast, for PMMC, as the fluid flows from the microchannel into the microchamber, the cross-sectional area increases, causing the fluid velocity to decrease, and the *Nu*_s_ value decreases accordingly. When the fluid flows from the merged chamber back into the MC, the sudden change in cross-sectional area causes the fluid to impinge on the ribs on both sides of the channel, leading to an increase in fluid velocity on both sides and a simultaneous increase in *Nu*_s_ values. At this point, the increase in *Nu*_s_ for PMMC-D is more significant. When the fluid reaches the next periodic merged microchamber, the same phenomenon occurs. This indicates that the vortex generator enhances momentum and heat exchange between the mainstream and near-wall fluids, thereby increasing *Nu*_s_ and boosting the heat transfer performance of this heat exchanger.

### 4.2. The Averaged Characteristics

#### 4.2.1. The Averaged Characteristics at Different Rib Widths, [*b*/*W*_ch_]

**Figure 11:**
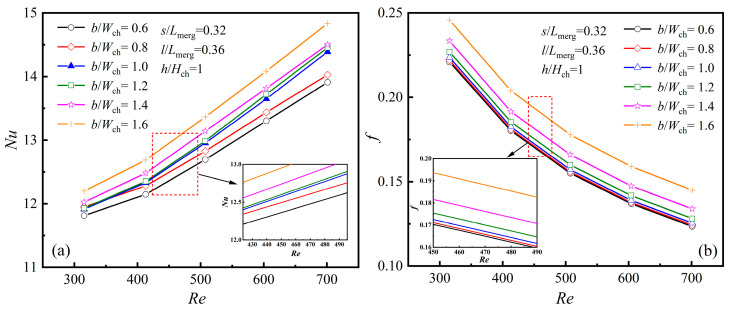
Effect of *b* at different *Re*s: (**a**) *Nu*; (**b**) *f*.

As illustrated in Figure 1, the diamond rib’s length is denoted by the parameter *l*, its width by *b*, its height by *h*, and its position by *s*. To further investigate the impact of diamond rib parameters on thermal performance and ensure the general applicability of the research findings, the parameters were nondimensionalized in this study, namely *h*/*H*_ch_, *b*/*W*_ch_, *l*/*L*_merg_, and *s*/*L*_merg_. When *s*/*L*_merg_ = 0.32, *l*/*L*_merg_ = 0.36, and *h*/*H*_ch_ = 1, the variations in the average *Nu* and friction factor *f* with *Re* under different *b*/*W*_ch_ values are shown in Figure 11a,b.

As illustrated in Figure 11a,b, both *Nu* and *f* rise as *b*/*W*_ch_ increases. When *b*/*W*_ch_ = 1.6, the *Nu* value reaches its maximum, indicating optimal heat transfer performance at this point, though it is accompanied by a notable increase in *f*. This indicates that increasing the rib width enhances thermal performance while also resulting in higher flow resistance. The effect of rib width can be explained by the flow patterns around the diamond ribs shown in Figure 5. A wider rib reduces the flow channel between ribs, leading to an increase in fluid velocity as a result of the Bernoulli effect. Enhanced fluid mixing and a larger recirculation zone in the wake contribute to better heat transfer, but the narrowed channel results in higher flow resistance. It is noteworthy that when designing *b*/*W*_ch_, when *b*/*W*_ch_ increases, the distance between adjacent ribs gradually decreases, leading to a significant increase in channel flow blockage. This, in turn, results in a substantial rise in flow resistance. Therefore, the maximum value of *b*/*W*_ch_ is set to 1.6.

#### 4.2.2. The Averaged Characteristics at Different Rib Lengths, [*l*/*L*_merg_]

**Figure 12:**
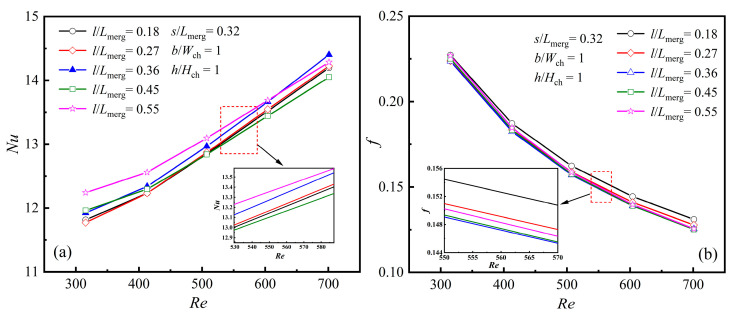
Effect of *l* at different *Re*s: (**a**) *Nu*; (**b**) *f*.

As illustrated in Figure 12a, *Nu* increases as *Re* rises. When *Re* < 600, the highest *Nu* is observed when the rib length *l*/*L*_merg_ is 0.55. For a constant rib width, changing the rib length enlarges the heat transfer surface and enhances the *Nu* value. However, increasing the rib length reduces the separation angle. A smaller separation angle results in a reduction in the transverse velocity of the fluid, thereby diminishing the heat transfer efficiency. When *Re* > 600, the maximum *Nu* is achieved when the rib length *l*/*L*_merg_ is 0.36.

In Figure 12b, *f* gradually decreases as *Re* increases. For a fixed rib width, extending the rib length brings the rib’s tip closer to the inlet of the merging chamber, causing earlier mainstream separation. At the same time, a smaller separation angle reduces the transverse velocity, thereby decreasing flow resistance. Therefore, a longer rib does not always guarantee better thermal performance. While increasing the rib length alters the heat transfer surface area, it also affects the separation angle. A larger separation angle increases transverse velocity, promotes boundary layer redevelopment, and improves thermal performance. Thus, the selection of rib length requires comprehensive consideration. This also explains the trend of *Nu* in Figure 12a, especially when *Re* > 600 and the rib length *l*/*L*_merg_ is 0.36.

#### 4.2.3. The Averaged Characteristics at Different Rib Heights, [*h*/*H*_ch_]

**Figure 13:**
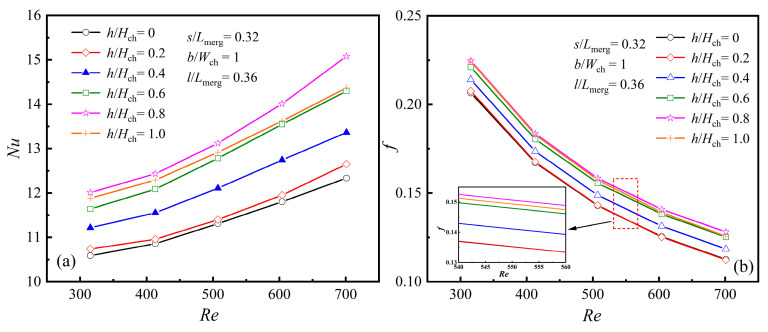
Effect of *h* at different *Re*s: (**a**) *Nu*; (**b**) *f*.

When the rib parameters are set as *s*/*L*_merg_ = 0.32, *b*/*W*_ch_ = 1, and *l*/*L*_merg_ = 0.36, the changes in *Nu* and *f* with *Re* for various values of *h*/*H*_ch_ are shown in Figure 13a,b. Here, *h*/*H*_ch_ = 0 indicates that no diamond rib structure is present in the merging chamber, while *h*/*H*_ch_ = 0.2 indicates that the rib height equals the height of the microchannel.

As illustrated in Figure 13a,b, when *Re* remains unchanged, both *Nu* and *f* generally increase with the rise in *h*/*H*_ch_. However, a turning point occurs when *h*/*H*_ch_ = 0.8. When *h*/*H*_ch_ = 0.8, both *Nu* and *f* reach their maximum values, indicating that the rib achieves optimal heat transfer performance at this height, albeit with the highest flow resistance. Figure 13b also shows that when *h*/*H*_ch_ < 0.4, the rib height is relatively low, resulting in an insignificant impact on *f*.

#### 4.2.4. The Averaged Characteristics at Different Rib Mounted Positions, [*s*/*L*_merg_]

**Figure 14:**
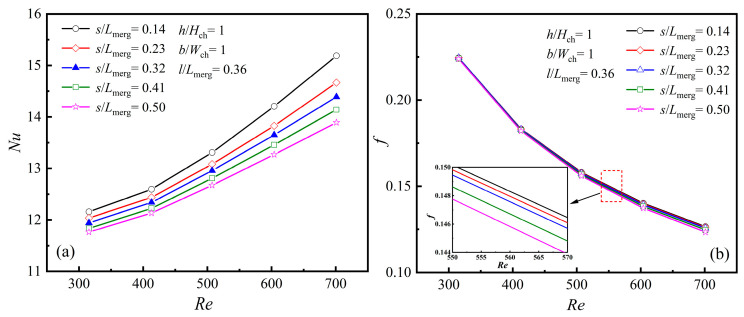
Effect of *s* at different *Re*s: (**a**) *Nu*; (**b**) *f*.

The position parameter of the diamond rib in the merging chamber is represented by *s*/*L*_merg_, which denotes the distance from the front end of the merging chamber to the diamond rib. By varying the value of *s*/*L*_merg_, the heat dissipation performance of the rib at different positions relative to the front end of the merging chamber can be determined. When *h*/*H*_ch_ = 1, *b*/*W*_ch_ = 1, and *s*/*L*_merg_ = 0.36, the changes in *Nu* and *f* with *Re* for various values of *s*/*L*_merg_ are presented in Figure 14a,b.

As illustrated in Figure 14a, when *Re* is constant, the *Nu* value gradually decreases with an increase in *s*/*L*_merg_, and the rate of decrease becomes more pronounced as *Re* increases. A smaller *s*/*L*_merg_ indicates that the rib is closer to the front end of the merging chamber. When the fluid flows from the MC into the expanded merging chamber, the time for the fluid to impact the rib is shortened, leaving insufficient time for the fluid to decelerate. Instead, this causes local acceleration of the mainstream separation, thereby enhancing the *Nu* value. In this case, the upstream vortex region decreases, while the downstream vortex region increases. As *s*/*L*_merg_ increases, the upstream vortex region grows larger, and the fluid gradually decelerates and expands after entering the suddenly expanded merging chamber. The time for the fluid to impact the rib becomes longer, resulting in a slightly prolonged fluid separation time and reduced velocity, which lowers the average *Nu* value.

As illustrated in Figure 14b, *f* gradually declines with rising *Re* as the position parameter s/Lmerg changes. When *Re* < 400, *s*/*L*_merg_ has little effect on *f*. However, when *Re* > 400, the influence of *s*/*L*_merg_ on *f* gradually increases as fluid velocity and pressure drop rise. With the increase in *s*/*L*_merg_, the fluid decelerates and expands after entering the suddenly expanded merging chamber, resulting in a reduction in velocity and a gradual decrease in *f*.

#### 4.2.5. The Correlations of *Nu* and *f*

The correlations of *Nu* and *f* are crucial for the application of diamond ribs in MC design. To derive these correlations, a multiple non-linear regression analysis is performed to relate *Nu* and *f* with *Re*, *l*, *b*, *h*, and *s*, respectively.

(36) $$Nu=3.0Re^{0.232}{\left(\frac{b}{W_{\mathrm{ch}}}\right)}^{0.045}{\left(\frac{l}{L_{\mathrm{merg}}}\right)}^{0.015}{\left(\frac{h}{H_{\mathrm{ch}}}\right)}^{0.071}{\left(\frac{s}{L_{\mathrm{merg}}}\right)}^{-0.041}$$

(37) $$f=13.9Re^{-0.722}{\left(\frac{b}{W_{\mathrm{ch}}}\right)}^{0.125}{\left(\frac{l}{L_{\mathrm{merg}}}\right)}^{-0.021}{\left(\frac{h}{H_{\mathrm{ch}}}\right)}^{0.064}{\left(\frac{s}{L_{\mathrm{merg}}}\right)}^{-0.005}$$

(38) $$P_{Nu}=3.0{\left(\frac{b}{W_{\mathrm{ch}}}\right)}^{0.045}{\left(\frac{l}{L_{\mathrm{merg}}}\right)}^{0.015}{\left(\frac{h}{H_{\mathrm{ch}}}\right)}^{0.071}{\left(\frac{s}{L_{\mathrm{merg}}}\right)}^{-0.041}$$

(39) $$P_{f}=13.9{\left(\frac{b}{W_{\mathrm{ch}}}\right)}^{0.125}{\left(\frac{l}{L_{\mathrm{merg}}}\right)}^{-0.021}{\left(\frac{h}{H_{\mathrm{ch}}}\right)}^{0.064}{\left(\frac{s}{L_{\mathrm{merg}}}\right)}^{-0.005}$$

where 315 ≤ *Re* ≤ 701, 0.6 ≤ *b*/*W*_ch_ ≤ 1.6, 0.18 ≤ *l*/*L*_merg_ ≤0.55, 0.2 ≤ *h*/*H*_ch_ ≤ 1.0, 0.14 ≤ *s*/*L*_merg_ ≤ 0.5.

**Figure 15:**
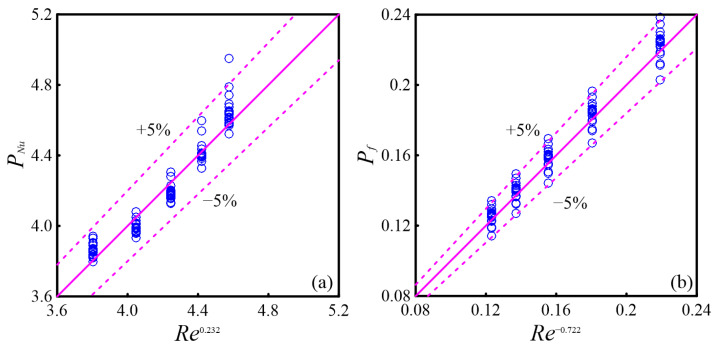
Comparison of the numerical results with the fitting formula: (**a**) *Nu*; (**b**) *f*.

The fitting of Equations (38) and (39) to the numerical data is shown in Figure 15, with maximum deviations for *Nu* and *f* both under 5%. According to Equation (38), the position parameter *s* of the diamond rib negatively affects *Nu*, while other parameters contribute positively. Equation (39) indicates that the width *b* is the main geometric factor influencing the friction coefficient *f*.

### 4.3. Heat Transfer Enhancement and Its Mechanisms

#### 4.3.1. Heat Transfer Enhancement

- Heat transfer enhancement at different rib widths, [*b*/*W*_ch_]

**Figure 16:**
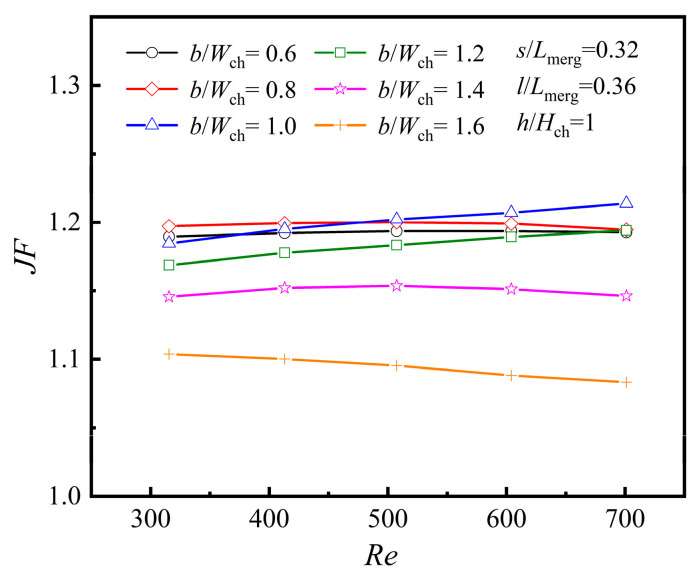
Effect of *b* at different *Re*s.

To fully assess the influence of diamond rib characteristics on the heat transfer efficiency of MCs, both heat transfer and flow resistance need to be taken into account. Therefore, *JF* can be used as a comprehensive evaluation metric, representing the degree of improvement in heat transfer under constant mass flow conditions. A *JF* magnitude exceeding 1 signifies that the benefits of enhanced thermal transfer surpass the pressure losses resulting from fluid flow across the ribs. Furthermore, as indicated by Equation (27), a higher *JF* corresponds to better overall efficacy of the MC. Figure 16 illustrates the changes in *JF* with Re for various *b*/*W*_ch_ configurations.

The numerical results in Figure 16 indicate that when *Re* ≤ 500, *JF* reaches its maximum value at *b*/*W*_ch_ = 0.8. However, when 500 < *Re* < 700, the maximum *JF* occurs at *b*/*W*_ch_ = 1.0, and *JF* exhibits an approximately linear positive growth trend as *Re* increases. Although the rib width of *b*/*W*_ch_ = 1.6 provides the highest heat transfer performance, its *JF* value is the smallest, suggesting that the rise in frictional pressure drop from flow blockage exceeds the gains from enhanced heat transfer. Therefore, the rib width should be optimized based on the range of *Re* to balance performance and frictional losses.

- Heat transfer enhancement at different rib lengths, [*l*/*L*_merg_]

**Figure 17:**
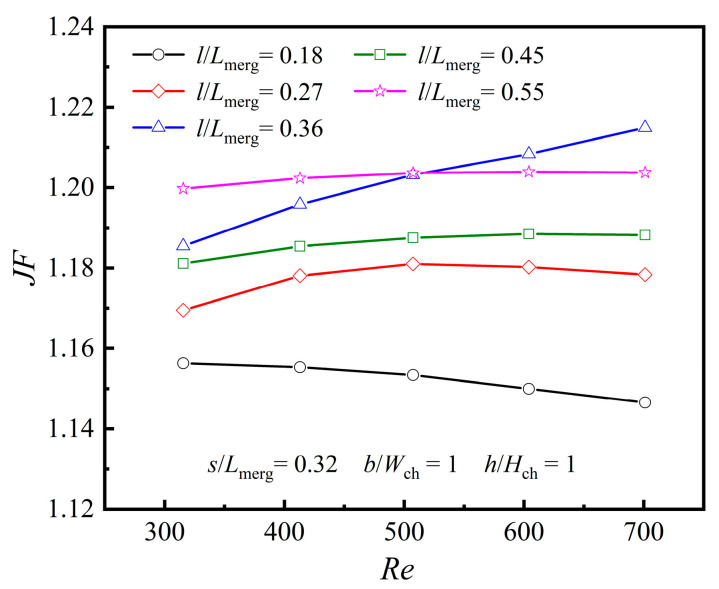
Effect of *l* at different *Re*s.

As shown in Figure 17, when *Re* ≤ 500, *JF* reaches its peak at *l*/*L*_merg_ = 0.55, while for 500 < *Re* < 700, the maximum *JF* occurs at *l*/*L*_merg_ = 0.36. For *Re* ≤ 500, the increase in the heat transfer surface has a more pronounced impact on improving heat transfer than the boundary layer redevelopment and secondary flow. However, when 500 < *Re* < 700, this effect is reversed, with boundary layer redevelopment and *Se* playing a more significant role in enhancing heat transfer.

- Heat transfer enhancement at different rib heights, [*h/H*_ch_]

**Figure 18:**
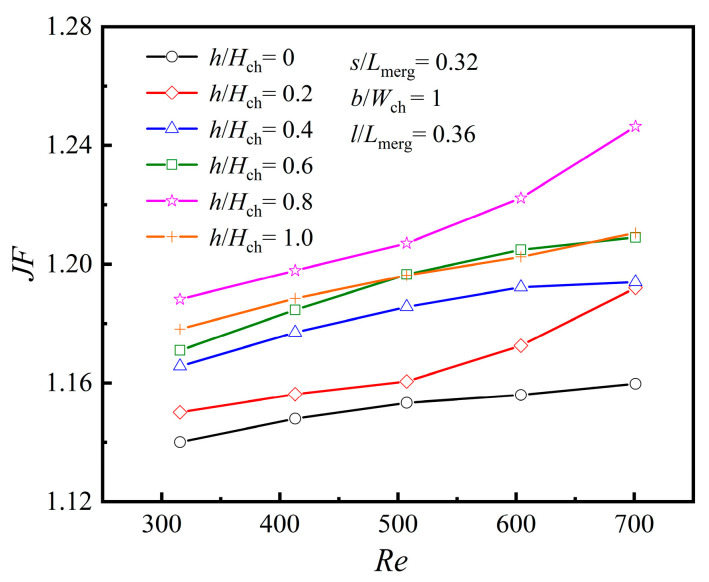
Effect of *h* at different *Re*s.

Figure 18 comprehensively analyzes the impact of rib presence and height on the heat dissipation performance of the MC. Figure 18 illustrates that *JF* increases progressively as *Re* rises. However, when the rib height *h*/*H*_ch_ = 1.0, *JF* does not reach its maximum value. Instead, the peak heat transfer performance is achieved at *h*/*H*_ch_ = 0.8, indicating that a higher rib height, equal to the MC height, does not necessarily yield the best results. When *h*/*H*_ch_ = 0, the channel corresponds to PMMC. Therefore, there is an optimal rib height that allows the diamond ribs to achieve the effect of enhanced heat transfer.

- Heat transfer enhancement at different rib mounted positions, [*s/L*_merg_]

**Figure 19:**
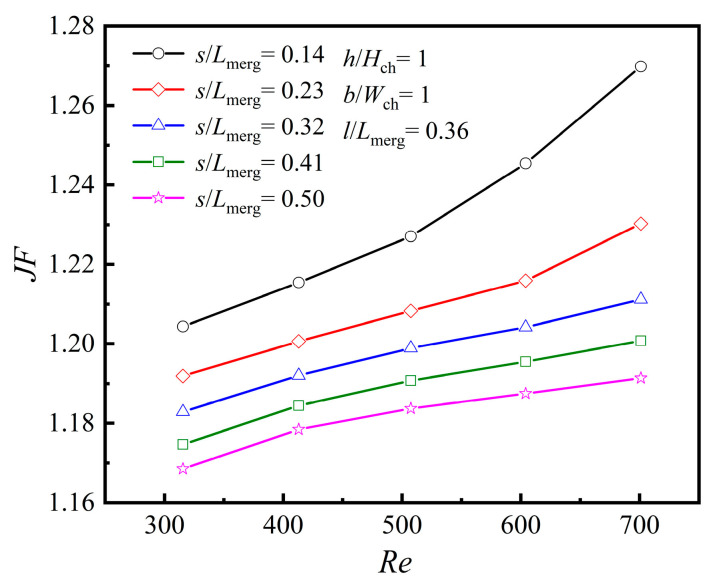
Effect of *s* at different *Re*s.

Figure 19 shows the variation in *JF* with *Re* for different *s*/*L*_merg_ values. It can be observed from the figure that as *s*/*L*_merg_ increases, *JF* decreases, while smaller *s*/*L*_merg_ values enhance *JF*, especially when *Re* > 400. In this case, the ribs improve fluid velocity and mixing, thereby enhancing heat transfer efficiency. As a result, in design refinement, *s* should be minimized as much as possible while taking manufacturing constraints into account.

The correlations of *JF*

(40) $$JF=1.02Re^{0.024}{\left(\frac{b}{W_{\mathrm{ch}}}\right)}^{-0.088}{\left(\frac{l}{L_{\mathrm{merg}}}\right)}^{0.036}{\left(\frac{h}{H_{\mathrm{ch}}}\right)}^{0.007}{\left(\frac{s}{L_{\mathrm{merg}}}\right)}^{-0.036}$$

where 315 ≤ *Re* ≤ 701, 0.6 ≤ *b*/*W*_ch_ ≤ 1.6, 0.18 ≤ *l*/*L*_merg_ ≤ 0.55, 0.2 ≤ *h*/*H*_ch_ ≤ 1.0, 0.14 ≤ *s*/*L*_merg_ ≤ 0.5.

**Figure 20:**
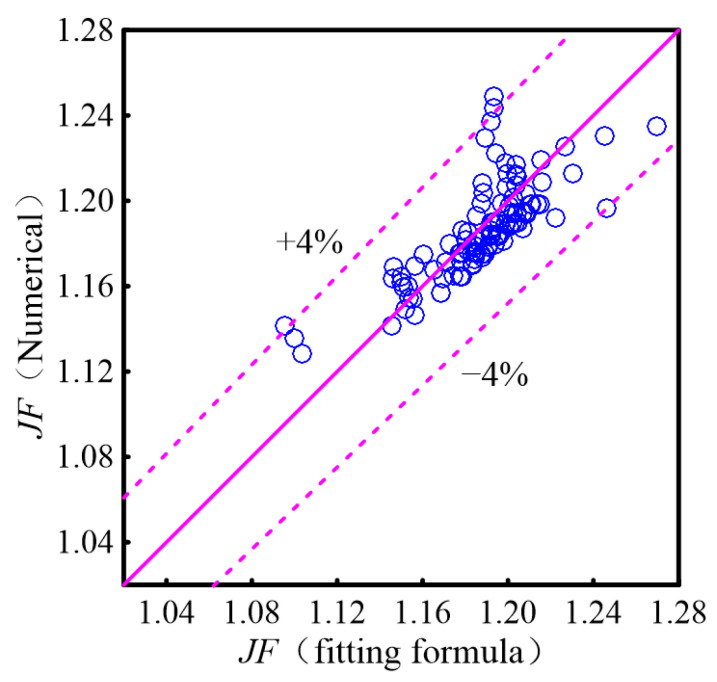
Comparison of the numerical results with the fitting formula.

Figure 20 presents a comparison between the fitting results of Equation (40) and the numerical calculation results. As illustrated, the fitting results for *JF* align well with the numerical data, with an error margin of less than 4%.

#### 4.3.2. Heat Transfer Enhancement Mechanism

**Figure 21:**
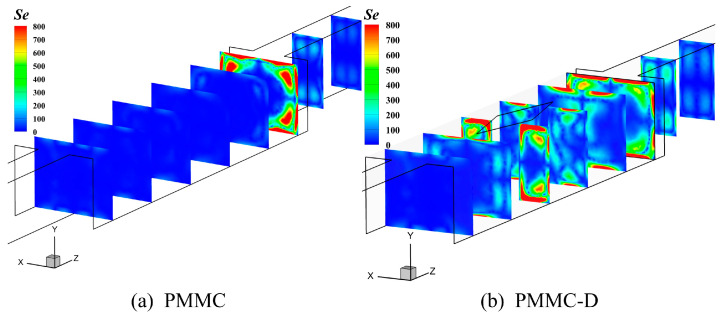
Secondary flow distributions and velocity fields on different cross-sections at *Re* = 500.

PMMC-D incorporates diamond-shaped ribs in the merging chamber, which enhance fluid perturbation and generate secondary flow intensity (*Se*) as the fluid passes through the diamond ribs, thereby effectively improving the heat transfer efficiency at the surface of the microchamber (MC). Secondary flow (*Se*), driven by the primary flow, refers to the flow occurring in a plane perpendicular to the primary flow direction [23,24]. It enhances fluid mixing within the microchamber (MC), disrupts the thermal boundary layer [25], and promotes momentum and thermal interaction between the mainstream and near-wall fluids, thereby improving the heat transfer efficiency and overall thermal performance of this type of heat sink. The use of diamond-shaped ribs is a key approach to generating secondary flow for heat transfer enhancement. The secondary flow field distributions of PMMC and PMMC-D are shown in Figure 21a,b.

As shown in Figure 21a,b, the secondary flow (*Se*) generated in the PMMC-D structure covers a significantly larger area compared to the PMMC structure. In PMMC, secondary flow only appears at the junction where the microchamber enters the microchannel (MC), which is caused by the constriction of the flow channel and the impingement of the fluid on both sides of the MC. In the PMMC-D structure, secondary flow is generated in the regions both upstream and downstream of the diamond ribs. When the fluid impacts the diamond ribs, the sharp and elongated structures at the front and rear ends allow the airflow to pass through smoothly. At the widest section of the ribs, the intensity of secondary flow is enhanced. Furthermore, the presence of the diamond ribs directs the surrounding fluid toward the wake region, disrupting the boundary layer in that area and generating secondary flow. Secondary flow also appears at the junction where the microchamber enters the MC. The comprehensive analysis demonstrates that the diamond ribs in PMMC-D effectively facilitate the momentum and thermal exchange between the mainstream fluid and the boundary layer flows, thereby enhancing the thermal performance of this type of heat sink.

**Figure 22:**
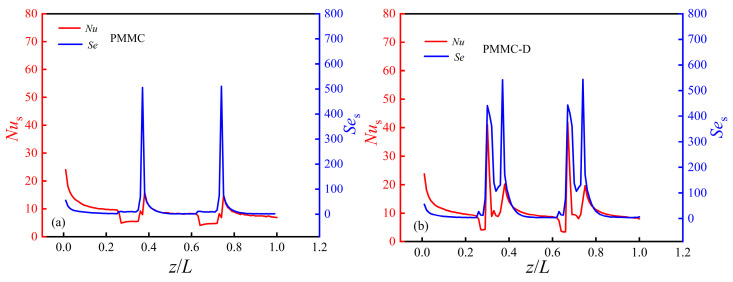
The span-averaged *Nu*_s_ and *Se*_s_ along the main flow direction: (**a**) PMMC; (**b**) PMMC-D.

Figure 22 presents the span-averaged local *Nu*_s_ and *Se*_s_ distributions along the main flow direction. Both metrics exhibit similar trends. At *z* = 0, the local *Nu*_s_ is high due to the entrance effect. As the liquid moves, the shear layer gradually thickens, and the entrance influence diminishes, leading to a decrease in *Nu*_s_. As shown in Figure 22a, In PMMC, when the fluid enters the expanded merging microchamber from the MC, the velocity decreases, resulting in a drop in *Nu*_s_. When the fluid enters the next constricted MC from the microchamber, it impacts the walls of the MC, causing a sudden increase in *Nu*_s_, which then drops again and stabilizes. *Se*_s_ follows the same trend.

As illustrated in Figure 22b, it is evident that in PMMC-D, when the fluid encounters the first diamond rib, *Nu*_s_ increases significantly, accompanied by a peak in *Se*_s_, indicating enhanced fluid disturbance and heat transfer. Similarly, when the fluid flows from the microchamber into the next constricted MC, it impacts the MC walls, resulting in another peak in *Nu*_s_ and *Se*_s_. At the location of the second diamond rib (*z* = 0.0065 m), *Nu*_s_ and *Se*_s_ increase significantly again, highlighting the periodic enhancement of thermal transfer in the ribbed areas. Notably, the diamond ribs in PMMC-D cause *Nu*_s_ and *Se*_s_ to exhibit two peaks within each microchamber. This study found that the average *Nu*_s_ and *Se*_s_ of the PMMC-D structure increased by 27.06% and 190.73%, respectively, compared to the PMMC structure.

### 4.4. Comparison with the Other Ribs

**Figure 23:**
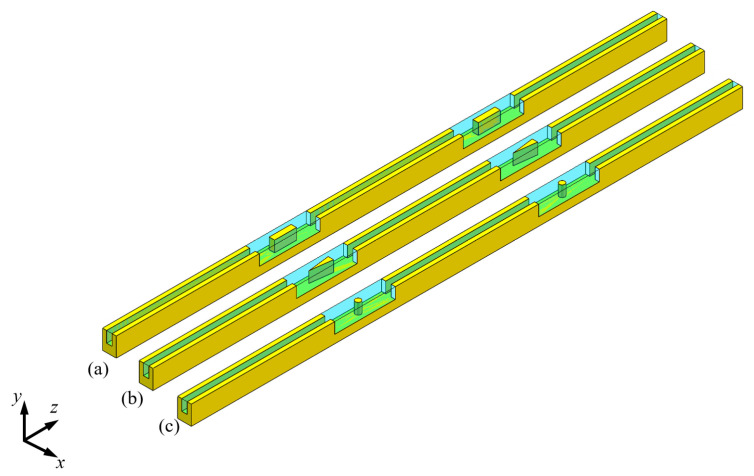
The other structures of MC: (**a**) periodically merged chamber rectangular MC mounted rectangular rib structure (PMMC-R) [13]; (**b**) PMMC mounted triangular rib structure (PMMC-T) [14]; (**c**) PMMC mounted cylindrical rib structure (PMMC-C) [12].

To evaluate the heat transfer performance of the MCs with the periodically merging chamber having diamond-shaped ribs, and to provide meaningful guidance for commonly used geometric configurations in industrial applications, this paper references the MCs with periodically merged chambers having rectangular ribs (PMMC-R) [13], triangular ribs (PMMC-T) [14], and cylindrical ribs (PMMC-C) [12] from the literature. Under the same basic conditions, the effects of different rib structures on flow resistance and heat transfer performance were calculated and compared. The different rib structures are shown in Figure 23.

**Figure 24:**
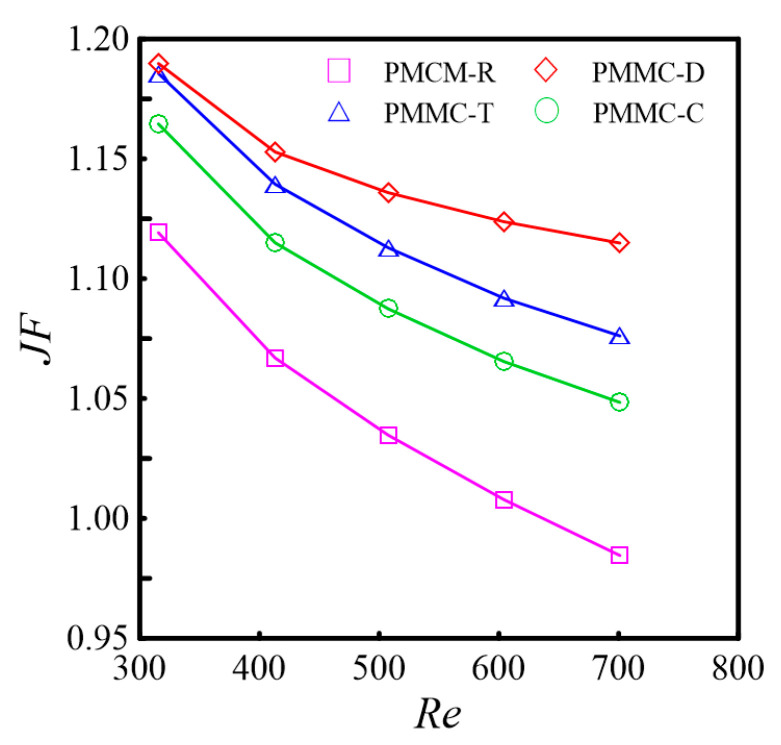
*JF* vs. *Re* of the different ribs.

The thermal transfer efficiency of these various rib shapes was analyzed and compared, with the calculation results presented in Figure 24.

As shown in Figure 24, the comprehensive performance indicator (*JF*) of PMMC-D is the highest. Within the studied range of *Re* values, the heat transfer enhancement factor decreases as the *Re* value increases. This indicates that at higher *Re* values, the pressure drop caused by flow resistance outweighs the benefits of enhanced heat transfer. Furthermore, as the *Re* value increases, the differences in heat transfer enhancement factors among different rib structures become more significant. The results show that for ribs with identical dimensions (rectangular, triangular, and diamond-shaped ribs with a width of 0.1 mm, length of 0.4 mm, and height of 0.2 mm, and cylindrical ribs with a radius of 0.1 mm and height of 0.2 mm), at *Re* = 700, the diamond-shaped ribs achieve enhancement effects of 3.59%, 13.24%, and 6.34% compared to triangular, rectangular, and cylindrical ribs, respectively, under the same mass flow rate. Through a comprehensive performance evaluation of these geometries, it is possible to assess performance trade-offs and determine optimal design criteria, providing meaningful guidance for commonly used geometric configurations in industrial applications.

## 5. Conclusions

This research utilizes numerical simulations to examine laminar flow and thermal transfer in MC heat sinks with periodic merging chambers and diamond ribs under constant wall heat flux. The key findings are as follows:

(1) Within the studied *Re* range, at *Re* = 700, diamond-shaped ribs (width 0.1 mm, length 0.4 mm, height 0.2 mm) achieve the highest heat transfer enhancement factor compared to triangular, rectangular, and cylindrical ribs. Specifically, they provide 3.59%, 13.24%, and 6.34% higher enhancement effects, respectively, under the same mass flow rate.

(2) The introduction of diamond rib structures in periodic merging chambers generates secondary flow, leading to complex shear layer dynamics and improved thermal transfer efficiency.

(3) The dimensional parameters of the diamond ribs were nondimensionalized. It was found that for the rib length *l*, the influence of *l*/*L*_merg_ on *Nu* and *f* varies with *Re*. When *Re* ≤ 500, the maximum heat transfer enhancement factor *JF* occurs at *l*/*L*_merg_ = 0.55; when 500 < *Re* < 700, it occurs at *l*/*L*_merg_= 0.36. Although increasing rib length expands the heat transfer area, its effect varies with *Re*. For lower *Re*, the increased area is more beneficial, whereas for higher Re, boundary layer effects dominate.

(4) For rib width *b*, the maximum *JF* occurs at *b*/*W*_ch_ = 0.8, when *Re* ≤ 500, and at *b*/*W*_ch_ = 1.0 when 500 < *Re* < 700. The maximum rib width of *b*/*W*_ch_ = 1.6 improves heat transfer but significantly increases friction, resulting in the lowest overall *JF*.

(5) The optimal rib height is found to be *h*/*H*_ch_ = 0.8, indicating that a full-height rib does not always yield the best performance.

(6) A smaller position parameter *s*/*L*_merg_ shortens the mainstream separation time, thereby improving heat transfer. Therefore, it is recommended to minimize the distance between the front end of the merging chamber and the rib, provided manufacturing allows for it.

## Figures and Tables

**Table 1:** Dimensions of the MC in Figure 1 (unit: mm).

*W*_b_	*W*_w_	*W*_ch_	*H*_ch_	*S*_z_	*L*_merg_	*l*	*h*	*b*	*s*	*L*_x_	*L*_y_	*L*_z_
0.15	0.3	0.1	0.2	3.7	1.1	0.4	0.2	0.16	0.35	2.5	0.35	10

**Table 2:** Thermal and physical properties of silicon.

*c*_p_ J/(kg·K)	*ρ* (kg/m^3^)	*λ* W/(m·K)
700	2330	148

**Table 3:** Verification of grid independence.

Scheme	Grid Number	${\bar{T}}_{f}$ (K)	*Nu*	*f*
1	1,280,165	297.8	11.04	0.16
2	1,064,070	297.8	10.99	0.15
3	886,439	297.8	10.83	0.15

## Data Availability

The original contributions presented in the study are included in the article, further inquiries can be directed to the corresponding authors.

## Nomenclature

*A*_b_	area of heated bottom wall [m^2^]
*A*_contact_	contact area between the fluid and the substrate [m^2^]
*b*	ribs width [m]
*C*_p_	specific heat [J/(kg·K)]
*D*_h_	hydraulic diameter [m]
*f*	average friction factor
*f*_app,ave_	Darcy friction coefficient
*h*	ribs height [m]
*h*_local_	local convective heat transfer coefficient [W/(m^2^.K)]
$\bar{h}$	average convective heat transfer coefficient [W/(m^2^.K)]
*H*_ch_	height of MCs [m]
*J* ^n^_ABS_	absolute vorticity [1/s]
*JF*	heat transfer performance factor
*Kn*	Knudsen number
*L*_x_, *L*_y_, *L*_z_	overall dimensions of the MC heat sink (width, height, length) [m]
*l*	ribs length [m]
*Nu*_local_	local Nusselt number
*Nu*	averaged Nusselt number
*Nu*_s_	span-averaged over the cross-sectional area
Δ*p*	pressure drop [Pa]
*q*	heat flux [W/m^2^]
*Re*	Reynolds number
*S*	distance of transverse MCs [m]
*s*	distance from the rib to the entrance of MC [m]
*Se*_v_	volumetrically aveaged dimensionless secondary flow strength
*Se*_s_	Cross-section averaged dimensionless secondary flow strength
*T*_f_	fluid temperature [K]
${\bar{T}}_{f}$	averaged fluid temperature [K]
*T*_w_	heated bottom wall temperature [K]
*U*_s_	characteristic velocity of secondary flow [m/s]
*w*_in_	fluid flow inlet velocity [m/s]
*w*_m_	average fluid flow velocity [m/s]
*W*_ch_	MC width [m]
*W*_w_	MC wall width [m]
*W*_b_	base thickness of the heat sink [m]
*x*,*y*,*z*	coordinates (Figure 1) [m]
Greeks	
$\alpha_{c}$	ratio of width to height of MC
*β*	separation angle
*k*	thermal conductivity [W/(m·K)]
*μ*	dynamic viscosity [kg/(m·s)]
*ρ*	density [kg/m^3^]
*ω*	vorticity component [1/s]
Subscripts	
ABS	absolute value
b	heat sink base
f	fluid
h	hydraulic
in	inlet
local	local value
m	mean
out	outlet
s	substrate, span averaged
0	rectangular straight MCs without microchambers
abbreviation list	
MC	micro channel
MEMS	micro electro-mechanical system
CMC	continuous micro channel
PMMC	MC having periodically merged chamber
PMMC-C	MC having periodically merged chamber mounted with cylindrical ribs
PMMC-D	MC having periodically merged chamber mounted with diamond ribs
PMMC-R	MC having periodically merged chamber mounted with rectangular ribs
PMMC-T	MC having periodically merged chamber mounted with triangular ribs
